# Andropause in Diabetic and Non-diabetic Males: A Cross-Sectional Observational Study in Western India

**DOI:** 10.7759/cureus.65152

**Published:** 2024-07-22

**Authors:** Ramiz S Kadiwala, Jagannath S Dhadwad

**Affiliations:** 1 General Medicine, Dr. D. Y. Patil Medical College, Hospital and Research Centre, Dr. D. Y. Patil Vidyapeeth (Deemed to be University), Pune, IND

**Keywords:** diabetes mellitus type 2, loss of libido, erectile dysfunction, male hypogonadism, andropause, low testosterone, diabetes

## Abstract

Andropause is defined as late-onset hypogonadism that increases with advancing age and is diagnosed based on symptoms of hypogonadism like loss of libido, loss of morning penile tumescence, and laboratory confirmation of low testosterone. Unlike menopause, it is a slow and progressive condition with varying symptoms and presentations. There is very little awareness and insufficient utilization of screening methods, and the majority of the cases remain undiagnosed. This study was done to get deeper insight into this topic and elicit correlations among different variables.

Objectives

The primary objective is to assess the prevalence of andropause in diabetic and non-diabetic males aged 40-60 years in the Maharashtra state of western India.

Material and methods

After ethics committee clearance, exclusion criteria were applied, and 120 participants were enrolled over a period of 21 months. All enrolled subjects were thoroughly evaluated for andropause symptoms. An early morning venous blood sample was taken and sent for routine blood tests, including HbA1c, serum total testosterone, and serum luteinizing hormone. Total testosterone values were compared in patients with symptoms of hypogonadism, loss of libido, and erectile dysfunction. The effects of HbA1c levels, duration of diabetes, body mass index (BMI), smoking, alcoholism, and hypertension on hypogonadism and low testosterone were assessed. Luteinizing hormone levels were compared among the case and control groups in subjects with low total testosterone.

Results

Total testosterone levels were low for age, loss of libido and erectile dysfunction were more common, and symptoms of hypogonadism appeared at an earlier age in diabetics compared to non-diabetics. The duration of diabetes and HbA1c had a negative impact on serum testosterone levels and andropause symptoms. Diabetic patients with low testosterone levels had significantly lower LH levels.

Conclusions

Andropause is a syndrome of hypogonadism that occurs due to low serum testosterone levels. This study puts emphasis on secondary hypogonadism playing an important role in diabetic patients, causing the early occurrence of andropause in them. Glycemic control and BMI have a significant effect on both andropause symptoms and total testosterone levels, necessitating strict glycemic control and lifestyle modifications to delay or prevent the occurrence of andropause.

## Introduction

Andropause, late-onset male hypogonadism, or male menopause are the terms used for hypogonadism symptoms occurring in males with advancing age. According to the Endocrine Society, male hypogonadism is a syndrome that results from the inability of the testis to produce physiological levels of testosterone and spermatozoa caused by disruption of the hypothalamic-pituitary-gonadal (HPG) axis [1]. The rate of decrease in testosterone levels varies among different individuals. The HPG axis is responsible for maintaining normal physiological levels of testosterone. The arcuate nucleus in the medial basal region of the hypothalamus is under negative control of testosterone and its metabolites; it secretes gonadotropin-releasing hormone (GnRH), sensing lower levels of testosterone. GnRH acts on the pituitary to secrete luteinizing hormone (LH), which acts on Leydig cells of the testis to produce testosterone. Hypogonadism can be primary or secondary. Primary or hypergonadotropic hypogonadism occurs due to testicular failure with low serum testosterone and high LH and follicle-stimulating hormone (FSH), caused by alcoholism, testicular injury, tumor or infection, Klinefelter syndrome, chemotherapy, or radiation treatment [2]. Secondary or hypogonadotropic hypogonadism is due to inadequate release of GnRH, and there is decreased LH and FSH, leading to insufficient stimulation of Leydig cells and low testosterone levels. It can be due to hypothalamic or pituitary lesions, hyperprolactinemia, Kallmann syndrome, or certain drugs. Alcohol can cause both primary and secondary hypogonadism [2].

Daily, up to 7 milligrams (mg) of testosterone secretion occur [3]. This is the usual target dose for testosterone replacement therapy (TRT). The secretion of LH is not continuous but pulsatile, with peaks every 90 to 120 minutes, with the highest levels being in the morning hours [4]. In older males, these bursts decrease [5]. LH is a glycoprotein hormone secreted from gonadotrophin cells of the anterior pituitary. It is an important hormone for maintaining the HPG axis. LH is under the negative feedback of testosterone. Testosterone is converted to estradiol in the brain; this estradiol acts on the pituitary to inhibit GnRH secretion. Apart from testosterone, dihydrotestosterone and estradiol can directly act on the pituitary to suppress the frequency of LH pulses [6]. LH contributes to the maturation of primordial germ cells and acts on interstitial Leydig cells of the testis to produce testosterone in males. LH stimulates steroidogenic acute regulatory (StAR) protein (rate-limiting step), cholesterol side-chain cleavage enzyme, and 17-alpha-hydroxylase to produce testosterone. This testosterone exerts its physiological functions. A fraction of this is converted to estradiol by aromatase or dihydrotestosterone by 5-alpha reductase. Both free testosterone and albumin-bound testosterone are termed bioavailable testosterone (BAT). Sex hormone-binding globulin (SHBG) plays an important role in keeping BAT in check. Therefore, total testosterone (TT) is a better indicator of BAT than free testosterone [7]. Functions of testosterone include maintaining reproductive tissues, stimulating and maintaining sexual function, stimulating spermatogenesis, increasing body weight by retention of nitrogen, improving lean body mass, promoting sebum production and body hair, and stimulating erythropoiesis [8]. Apart from this, testosterone also increases basal metabolic rate and improves mood and cognition [3,9].

Testosterone effects are vast; any derangement in its function causes great impairment in quality of life. The symptoms of low testosterone include decreased libido, increased adipose tissue, increased fatigue, low energy, low muscle mass, a depressed mood, and decreased sexual performance [8,10]. Diabetic patients are more likely to develop low testosterone levels and symptoms of hypogonadism. The rate of decline in testosterone is more profound and earlier when compared to non-diabetic subjects. There are studies showing hypogonadism symptoms are more frequent in diabetics, not only compared to same-age non-diabetics but also elderly non-diabetics [11].

In a developing country like India, there is less awareness regarding the complications of diabetes. These symptoms, which can be corrected with testosterone therapy, are often overlooked and considered manifestations of disease. In this study, we are comparing symptoms of hypogonadism with serum TT among diabetic and non-diabetic males and assessing the effects of uncontrolled hyperglycemia, duration of diabetes, and BMI on andropause.

## Materials and methods

This is a cross-sectional observational study done among 120 males aged 40-60 in the outpatient and inpatient departments of Dr. D. Y. Patil Medical College, Hospital and Research Center, Pune, India, from October 2022 to June 2024. Of 120 males, 80 patients were diagnosed with cases of type-2 diabetes mellitus (DM), and 40 were controls. The study received approval from the Institutional Ethics Committee of Dr. D. Y. Patil Medical College, Hospital and Research Center, Pune, with approval number IESC/PGS/2022/19, on September 28, 2022.

Inclusion criteria

Type-2 diabetic and non-diabetic males aged 40-60 years. A case should be a diagnosed type-2 DM patient with a duration of disease of at least seven years on treatment.

Exclusion criteria

Patients with chronic diseases like thyroid disorders, malignancies, and chronic diseases of the liver, kidney, and prostate were excluded. Patients with abnormal abdominal ultrasonography findings or with poor development of secondary sexual characteristics were also excluded. Subjects with a history of taking anti-psychotics, anti-depressants, beta-blockers, thiazides, anti-androgens, opiates, or recreational drugs were excluded. Unmarried men and those without their own biological children were excluded.

Data and sample collection

**Figure 1:**
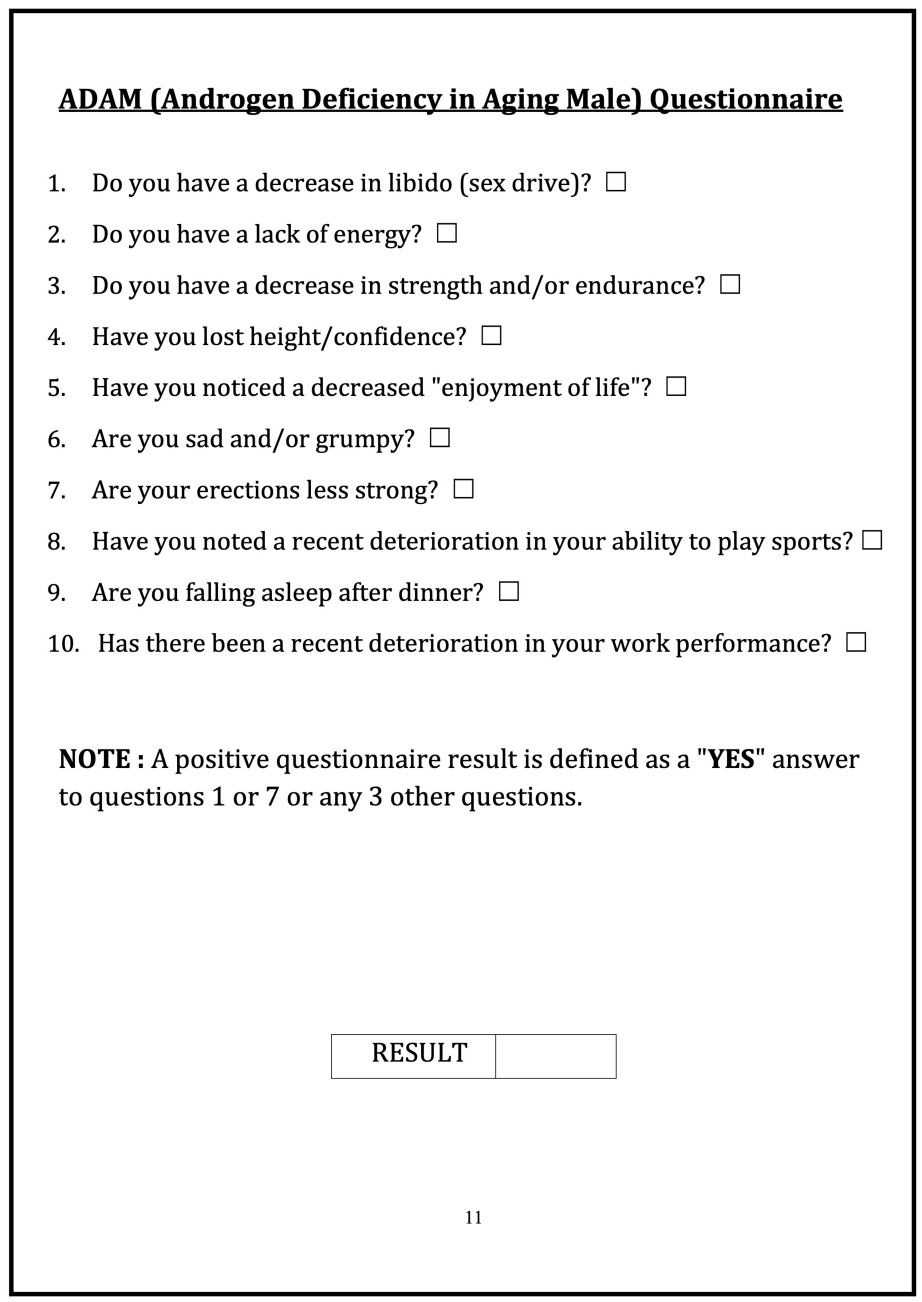
Androgen Deficiency in Aging Males (ADAM) questionnaire A positive questionnaire result was a "yes" answer to question one or seven or any three other questions.

Participants were selected based on non-probability purposive sampling. All demographic and patient data were collected with a questionnaire, a thorough history, and a review of the patient's past medical records. The Saint Louis University Androgen Deficiency in Aging Males (ADAM) questionnaire (Figure 1), curated by John E. Morley, after obtaining required permission for its use in educational degree-related research within the study titled "Comparative Study of Andropause in Type-2 Diabetic and Non-diabetic Males of Same Age Group" (13L/2022/18776), was given to each participant, and a score was given out of 10 [12]. Questions one and seven of the questionnaire were, “Do you have a decrease in libido (sex drive)?” and “Are your erections less strong?” A positive answer to any of these two questions or any other three out of eight questions that may include a decrease in muscle strength, loss of height, fatiguability, mood changes, or decreased work performance was considered ADAM positive, meaning they have symptoms of hypogonadism. The ADAM questionnaire has 60% specificity and 88% sensitivity [13]. After obtaining informed consent, enrolled subjects were evaluated, and ADAM score, body mass index (BMI), duration of diabetes, glycated hemoglobin (HbA1c), and LH levels were compared to TT levels in both diabetic and non-diabetic groups. 8 AM morning venous blood samples were sent for TT and LH levels to avoid any daytime variabilities. A TT value of less than 300 nanograms per milliliter (ng/dL) was considered low [14].

Statistical analysis plan

Data were entered in MS Excel and analyzed by Statistical Package for Social Sciences (SPSS) (IBM SPSS Statistics for Windows, IBM Corp., Version 26.0, Armonk, NY). Frequencies and percentages were interpreted for qualitative data. The mean and median (IQR) were assessed for quantitative data. The chi-square test and Fischer exact test were used to test the significance of categorical data. Continuous data appeared to have a skewed distribution; hence, the Mann-Whitney U test was applied to test the significance between categorical and continuous variables. The Spearman correlation was calculated between continuous variables. A p-value of less than 0.05 was considered statistically significant.

## Results

The overall age distribution of cases, controls, and all enrolled participants is given in Table 1.

**Table 1:** Overall age distribution in the study

Age	Diabetic cases		Controls		Overall	
	Frequency	Percentage	Frequency	Percentage	Frequency	Percentage
40-41	9	11.25%	6	15.00%	15	12.50%
42-43	8	10.00%	6	15.00%	14	11.67%
44-45	10	12.50%	7	17.50%	17	14.17%
46-47	12	15.00%	5	12.50%	17	14.17%
48-49	12	15.00%	5	12.50%	17	14.17%
50-52	6	7.50%	3	7.50%	9	7.50%
53-54	6	7.50%	3	7.50%	9	7.50%
55-56	4	5.00%	1	2.50%	5	4.17%
57-58	7	8.75%	2	5.00%	9	7.50%
59-60	6	7.50%	2	5.00%	8	6.67%
Total	80	100.00%	40	100.00%	120	100.00%

As shown in Table 2, there was a significant association between ADAM criteria being positive and diabetic subjects in groups A (p < 0.001) and B (p = 0.017). Also, for ADAM questions 1 (loss of libido) and 7 (erectile dysfunction), the p-values were significant, <0.001 and 0.004 for groups A and B, respectively.

**Table 2:** Association of ADAM criteria and ADAM question 1 or 7 positivity with cases and controls of different age groups Question 1 - Do you have a decrease in libido (sex drive)? Question 7 - Are your erections less strong? The comparison was made using Fisher's exact test. The p-value indicates probability; p < 0.05 means that the result is statistically significant, while p > 0.05 means that the result is not statistically significant. ADAM, Androgen Deficiency in Aging Males

Age-group	Subjects	ADAM criteria				ADAM question 1 or 7			
		Positive	Negative	Total	p-value	Positive	Negative	Total	p-value
Group A (40-49 years)	Cases	36 (70.6%)	15 (29.4%)	51	<0.001	20 (39.2%)	31 (60.8%)	51	<0.001
	Controls	0	29 (100.0%)	29		0	29 (100.0%)	29	
Group B (50-60 years)	Cases	29 (100%)	0	29	0.017	25 (86.2%)	4 (13.8%)	29	0.004
	Controls	8 (72.7%)	3 (27.3%)	11		4 (36.4%)	7 (63.6%)	11	

Comparing serum TT among cases and controls of both groups, p-values were significant for low serum testosterone in group B, while insignificant (p = 0.532) for group A (Table 3).

**Table 3:** Comparison of serum total testosterone levels in different age groups The comparison was made using Fisher's exact test. p < 0.05 is statistically significant. ng/dL - nanograms per deciliter

Serum total testosterone	Group A (40-49 years)		Group B (50-60 years)	
	Cases	Controls	Cases	Controls
<300 ng/dL	2 (3.9%)	0	23 (79.3%)	4 (36.4%)
>300 ng/dL	49 (96.1%)	29 (100.0%)	6 (20.7%)	7 (63.6%)
Total	51	29	29	11
p-value	0.532		0.020	

Among the diabetic subjects aged between 40 and 49 years, those with ADAM question 1 or 7 positive had a significantly longer duration of diabetes (8.05 years) and higher HbA1c (11.23). In the 50-60-year age group, patients with ADAM question 1 or 7 positive had significantly higher HbA1c of 12.77 (Table 4).

**Table 4:** Comparison of diabetes duration and HbA1c with ADAM question 1 or 7 positivity The Mann-Whitney U test is used for comparison; p < 0.05 is statistically significant. Question 1 - Do you have a decrease in libido (sex drive)? Question 7 - Are your erections less strong? ADAM, Androgen Deficiency in Aging Males; HbA1c - glycated hemoglobin

Age group	Parameter evaluated	ADAM question 1 or 7 positive	Number	Mean rank	Sum of ranks	p-value
Group A (40-49 years)	Duration of diabetes	Yes	20	35.88	717.50	<0.001
		No	31	19.63	608.50	
		Total	51	-	-	
	HbA1c	Yes	20	59.55	1,191.00	<0.001
		No	60	34.15	2,049.00	
		Total	80	-	-	
Group B (50-60 years)	Duration of diabetes	Yes	25	16.02	400.50	0.109
		No	4	8.63	34.50	
		Total	29	-	-	
	HbA1c	Yes	29	24.34	706.00	<0.001
		No	11	10.36	114.00	
		Total	40	-	-	

In Table 5, we have compared the duration of diabetes and HbA1c in diabetic patients with TT levels.

**Table 5:** Association of diabetes duration and HbA1c with serum total testosterone levels The Mann-Whitney U test is used for comparison. p < 0.05 is statistically significant. ng/dL - nanogram per deciliter

Age group	Parameter evaluated	Serum total testosterone	Number	Mean rank	Sum of ranks	p-value
Group A (40-49 years)	Duration of diabetes	<300 ng/dL	2	48.50	97.00	0.026
		>300 ng/dL	49	25.08	1,229.00	
		Total	51	-	-	
	HbA1c	<300 ng/dL	2	74.50	149.00	0.023
		>300 ng/dL	78	39.63	3,091.00	
		Total	80	-	-	
Group B (50-60 years)	Duration of diabetes	<300 ng/dL	23	16.20	372.50	0.142
		>300 ng/dL	6	10.42	62.50	
		Total	29	-	-	
	HbA1c	<300 ng/dL	27	24.37	658.00	0.003
		>300 ng/dL	13	12.46	162.00	
		Total	40	-	-	

When we applied the Mann-Whitney U test to the subjects with low testosterone (<300 ng/dL), diabetic subjects had significantly lower LH compared to the control group (p < 0.001), as shown in Table 6.

**Table 6:** Comparison of Luteinizing hormone in cases and control with testosterone less than 300 ng/dL A comparison between the groups was made using the Mann-Whitney U test. *Test cannot be performed on empty groups.

Age group	Subjects with total testosterone <300 ng/dL	Number	Mean rank	Sum of ranks	p-value
Group A (40-49 years)	Cases	2	1.50	3.00	Not valid*
	Controls	0*	0.00	0.00	
	Total	2		-	-
Group B (50-60 years)	Cases	23	12.00	276.00	<0.001
	Controls	4	25.50	102.00	
	Total	27	-	-	-

The Mann-Whitney U test showed a significant association of higher BMI with low testosterone <300 ng/dL (p = 0.032) and andropause symptoms (p < 0.001), as shown in Table 7.

**Table 7:** Effects of BMI on serum total testosterone levels and ADAM criteria p <0.05 is statistically significant. A comparison between the groups was done using the Mann-Whitney U test. ADAM, Androgen Deficiency in Aging Males

Comparison with BMI	Value	Number	Mean rank	Sum of ranks	p-value
Serum total testosterone	<300 ng/dL	29	72.55	2,104.00	0.032
	>300 ng/dL	91	56.66	5,156.00	
ADAM criteria	Positive	73	74.70	5,453.00	<0.001
	Negative	47	38.45	1,807.00	

Common comorbidities and common addictions were also evaluated for testosterone levels and symptoms of hypogonadism. The effects of hypertension, alcoholism, and smoking on serum TT and andropause symptoms were evaluated. No significant effect of alcoholism, smoking, or hypertension on serum testosterone levels or ADAM criteria was found among the enrolled subjects, as shown in Table 8.

**Table 8:** Comparison of the effects of hypertension, alcoholism, and smoking on ADAM criteria and serum total testosterone levels The comparison was made using the chi-square test. p > 0.05 is statistically not significant. ADAM, Androgen Deficiency in Aging Males

Parameter	Result	Hypertensive			Alcoholic			Smoker		
		Yes	No	Total	Yes	No	Total	Yes	No	Total
ADAM criteria	Positive	16 (66.7%)	57 (59.4%)	73 (60.8%)	31 (60.8%)	42 (60.9%)	73 (60.8%)	15 (57.7%)	58 (61.7%)	73 (60.8%)
	Negative	8 (33.3%)	39 (40.6%)	47 (39.2%)	20 (39.2%)	27 (39.1%)	47 (39.2%)	11 (42.3%)	36 (38.3%)	47 (39.2%)
	p-value	0.513			0.992			0.711		
Serum total testosterone	<300 ng/dL	9 (37.5%)	20 (20.8%)	29 (24.2%)	12 (23.5%)	17 (24.6%)	29 (24.2%)	7 (26.9%)	22 (23.4%)	29 (24.2%)
	>300 ng/dL	15 (62.5%)	76 (79.2%)	91 (75.8%)	39 (76.5%)	52 (75.4%)	91 (75.8%)	19 (73.1%)	72 (76.6%)	91 (65.8%)
	p-value	0.088			0.889			0.710		

## Discussion

In the results section, we showed that symptoms of andropause were more prevalent and were seen at an earlier age when compared to the control group. Around 70.60% of diabetic subjects (n = 51) in the age group 40-49 had ADAM criteria positive, compared to none in the control group (n = 29), while 39.20% of diabetic subjects had complaints of loss of libido and erectile dysfunction compared to none in the control group. In the 50-60 age group, all diabetic patients (n = 29) had symptoms of andropause, compared to 72.7% in the control group (n = 11), while 86.20% of diabetic subjects had loss of libido and erectile dysfunction compared to just 36.40% in the control group. In the 50-60 age group, 79.30% of diabetes cases had a marked decline in testosterone compared to 36.40% in the control group, meaning there is a significantly higher occurrence of low testosterone in diabetic subjects compared to normal individuals. This is consistent with many other studies showing a diabetes association with early hypogonadism and low testosterone levels [15-17]. We compared the duration of diabetes and HbA1c in diabetic patients with symptoms of loss of libido and erectile dysfunction. The duration of diabetes and glycemic control had a significant impact on the loss of libido and erectile dysfunction. Among the diabetic subjects aged between 40 and 49 years, those with symptoms had a significantly longer duration of diabetes (8.05 years) and higher HbA1c (11.23), while the 50-60-year age group had a significantly higher HbA1c of 12.77. Similar results were found in the comparison of low total testosterone levels (<300 ng/dL) with the duration of diabetes and HbA1c.

LH levels are very important for the normal functioning of the testis and testosterone physiology, as previously described. LH levels normally increase with age to compensate for low testosterone in old age [17]. In our study, diabetics in the 50-60-year age group with low testosterone had significantly lower LH levels compared to non-diabetics. Similar to our results, Hermann et al. found that in diabetic patients, decreased testosterone levels did not upregulate sufficient pituitary gonadotropin secretion [17]. Hayek et al. also concluded in their study that compensatory increases in LH were absent or inadequate, causing relatively lower levels of LH and secondary hypogonadism in DM compared to the control group [18]. There may be a subtle defect in pituitary up-regulation that is contributing to decreased testicular function. BMI is an important factor for diabetic patients, and often, a higher BMI correlates with more instances of diabetes-related complications. In our study, we compared serum total testosterone levels and symptoms of hypogonadism with BMI. BMI was significantly higher among the patients with low serum total testosterone (26.91 kg/m^2^) and patients with andropause symptoms (27.17 kg/m^2^). This finding is in line with other studies showing a negative correlation between BMI and testosterone levels [19]. 

There is consistent evidence that low testosterone and diabetes mellitus are closely interconnected. The Endocrine Society also recommends routine serum testosterone measurements in all diabetic patients [1]. This would be beneficial for diabetic patients suffering from hypogonadal symptoms but unaware of this entity. Simple testosterone replacement therapy (TRT) would be enough to improve their quality of life. Until now, TRT was prescribed very cautiously, with a probable risk of increased cardiovascular events. Recently, a multicenter, randomized, large-scale, double-blind trial on the cardiovascular safety profile of TRT showed no significant increase in cardiovascular events compared to placebo [20]. This would definitely put treating physicians at ease when starting TRT.

Limitations

We have conducted an analytical study and determined the association, though the causality of lower testosterone could not be ascertained due to study design limitations. Many diabetic cases were evaluated on an inpatient basis, while most of the controls were enrolled on an outpatient basis. This can have an effect on the perception of subjective aspects, like questions regarding hypogonadism symptoms, erectile dysfunction, and loss of libido. Also, the microvascular complications of diabetes and their effect on hypogonadism symptoms and low testosterone were not evaluated separately.

## Conclusions

Andropause prevalence increases with advancing age, but in diabetic patients, it occurs earlier than in normal individuals, and symptoms are more severe. Loss of libido and erectile dysfunction are seen earlier in diabetic patients. The duration of diabetes, glycemic control, and BMI have a significant impact on andropause, as poorer glycemic control and higher BMI are associated with more hypogonadism symptoms and lower testosterone levels at an earlier age. All diabetic patients should be evaluated for andropause more routinely, more awareness should be spread, tighter glycemic control should be maintained, and informed judgment should be taken on whether to start early TRT to improve their quality of life.
